# The Role of *Gynostemma pentaphyllum* Extract in Combating H_2_O_2_‐Induced Oxidative Stress and Preventing Hair Graying

**DOI:** 10.1155/drp/1775262

**Published:** 2026-06-08

**Authors:** Xiaojin Liu, Lei Chang, Yaqian Qiu, Xiaobing Lv, Tiancheng Ji

**Affiliations:** ^1^ Anhui Provincial Key Laboratory of Molecular Enzymology and Mechanism of Major Metabolic Diseases, College of Life Sciences, Anhui Normal University, Wuhu, 241000, Anhui, China, ahnu.edu.cn; ^2^ Anhui Provincial Engineering Research Centre for Molecular Detection and Diagnostics, College of Life Sciences, Anhui Normal University, Wuhu, 241000, Anhui, China, ahnu.edu.cn

**Keywords:** *Gynostemma pentaphyllum* (Thunb.) Makino extract, hair graying, melanocytes, oxidative stress, Wnt/β-catenin signaling pathway

## Abstract

**Objectives:**

Hair graying, often linked to aging (oxidative stress), has gained significant attention due to its social implications and the desire for a youthful appearance. Currently, there is no effective solution. The objective of this study was to determine whether *Gynostemma pentaphyllum* (Thunb.) Makino extract (GP) can mitigate the effects of H_2_O_2_‐induced oxidative stress in melanocytes and evaluate its potential as a treatment for hair graying.

**Methods:**

Oxidative stress models were established using B16 melanoma cells and C57BL/6 mice. GP treatment’s effects on cell viability, melanocyte apoptosis, intracellular reactive oxygen species (ROS) levels, melanin production, tyrosinase activity, total superoxide dismutase (SOD) activity, and the Wnt/β‐catenin signaling pathway were assessed. In animal experiments, the protective effects of GP on mouse hair follicles were evaluated.

**Results:**

GP treatment enhanced cell viability, decreased melanocyte apoptosis, reduced intracellular ROS levels, increased melanin production, boosted tyrosinase and SOD activity, and potentially modulated the Wnt/β‐catenin signaling pathway. In animal experiments, GP significantly protected mouse hair follicles from H_2_O_2_‐induced damage.

**Conclusion:**

The findings suggest that GP has potential as a treatment for hair graying by mitigating H_2_O_2_‐induced oxidative stress in melanocytes, highlighting its therapeutic potential in addressing oxidative stress‐related hair graying.

## 1. Introduction

The hair, an emblematic feature of human appearance, undergoes a cyclical process of growth, regression, and rest, known as the hair growth cycle [1, 2]. The process of hair graying, a phenomenon often associated with aging, has garnered significant attention due to its social implications and the desire to maintain a youthful appearance [3]. While various strategies have been developed to address or conceal graying hair, including the use of hair dyes and herbal remedies, scientific evidence supporting their effectiveness remains limited [4]. At the heart of the hair pigmentation process lies the melanocyte, a specialized cell located in the hair follicle bulb responsible for producing pigment. Melanocytes synthesize melanin, the pigment that imparts color to hair, through a series of enzymatic reactions involving enzymes like tyrosinase [5, 6]. Subsequently, melanin is transferred to adjacent keratinocytes, where it becomes incorporated into the growing hair shaft, giving it its characteristic color [7]. Thus, the regulation of melanocyte activity is crucial for maintaining normal hair pigmentation throughout one’s lifetime [8].

Recent findings suggest that oxidative stress also plays a significant role in promoting age‐induced hair graying [6, 9, 10]. Oxidative stress, defined by an imbalance between the production of reactive oxygen species (ROS) and the body’s antioxidant defense mechanisms, can cause damage to biomolecules and cellular structures, leading to cellular dysfunction and death [11]. Oxidative stress induced by ROS, such as hydrogen peroxide (H_2_O_2_), inhibits melanocyte proliferation and melanin production, contributing to hair graying [8, 11, 12].

*Gynostemma pentaphyllum* (Thunb.) Makino (GP), belonging to the family *Cucurbitaceae* and the genus *Gynostemma*, is a herbaceous climbing plant, was first recorded in the “Compendium of Materia Medica for Famine Relief” published in China in 1404 [13]. In the view of traditional Chinese medicine, GP is used for the treatment of cardiovascular disease [14, 15] and anxiety neurosis [8, 16], protecting the liver, promoting sleep, and treating gastrointestinal inflammation, bronchitis, and pharyngitis [17]. Although our previous work suggested GP may exert antigraying effects, no detailed mechanistic studies have comprehensively evaluated its protective role against oxidative stress–induced hair graying.

In this study, we explored the effects of GP on preventing hair graying both in vitro and in vivo, utilizing B16 cell cultures and the C57BL/6 mouse model under H_2_O_2_‐induced stress. Our previous research demonstrated that GP effectively promotes hair growth [8]. Combined with the current findings, these results underscore GP’s significant role in promoting hair growth and inhibiting hair graying. Consequently, GP has potential applications in pharmaceutical and cosmetic products aimed at enhancing hair health.

## 2. Materials and Methods

### 2.1. Reagents

B16 melanoma cells were cultured in Dulbecco’s Modified Eagle’s Medium (DMEM) (Hyclone, UT) supplemented with 10% fetal bovine serum and 1% penicillin–streptomycin (Gibco). The Cell Counting Kit‐8 (CCK‐8) was obtained from Uelandy (Shanghai, China). The Total Superoxide Dismutase Assay Kit with WST‐8 was acquired from Beyotime (Shanghai, China). GP was sourced from Shanghai Zhina Biotechnology Co., Ltd. The extract was prepared from the aerial parts (leaves and stems) of GP. C57BL/6 mice were purchased from SCBS Biotechnology Co., Ltd. (Henan, China). Paraffin was procured from China Pharmaceutical Group Chemical Reagent Co., Ltd.

### 2.2. Cell Vitality and Proliferation Assay

Cell vitality and proliferation were assessed using a CCK‐8 reagent [8], which is widely used for sensitive and quantitative assessment of cell viability. Briefly, B16 melanoma cells were seeded into 96‐well plates (5 × 10^3^ cells/well) and treated with GP at the indicated concentrations for 24 h. CCK‐8 reagent (10 μL) was then added to each well and incubated for 2 h at 37°C, and absorbance was measured at 450 nm.

### 2.3. Determination of Melanin Content

B16 cells (2.5 × 10^5^ cells per 35 mm dish) were sorted into three distinct experimental groups: the control group, exposed to H_2_O_2_ (1.5 mM) and subjected to a combination of H_2_O_2_ exposure and GP treatment. Following treatment, cells underwent digestion, centrifugation, and subsequent imaging. Subsequently, cell lysis was performed using 100 μl of 1 mol/L NaOH in a water bath set at 60°C for 1 h. Absorbance of the lysed cells was then measured at 405 nm using a microplate reader (TECAN Spark 10 M, USA).

### 2.4. Measurement of Intracellular Tyrosinase and Total Superoxide Dismutase (SOD) Activity

The tyrosinase activity assay followed Ramsden’s method [18]. Samples were dissolved in DMSO and utilized at a 100‐fold dilution for the experiment. A mixture comprising 0.1‐M potassium phosphate buffer (pH 6.8), 3‐mM L‐tyrosine solution with or without the sample chemical, and 2000 units/mL tyrosinase in aqueous solution was prepared. Following a 10‐min incubation at 37°C, the reaction was monitored by measuring the absorbance at 475 nm.

For the detection of SOD activity, an SOD assay kit (WST‐1 method) was employed. Total proteins were extracted from B16 cells according to the manufacturer’s instructions. The activities of SOD in the samples were then measured at a wavelength of 450 nm using a microplate reader. SOD activity was presented as a percentage relative to the untreated control group (=100%).

### 2.5. Flow Cytometry Was Employed to Investigate ROS Levels

B16 cells were seeded into a 6‐well plate, with 1 mL of cell suspension added to each well. The 6‐well plate was placed in a cell incubator and cultured for 24 h. After incubation, the medium was discarded, and the wells were washed with PBS. Each well was then treated with 1.5 mM H_2_O_2_ for 30 min, followed by discarding the H_2_O_2_ solution. Different concentrations of GP were added to the wells, and the cells were cultured for 48 h. Following treatment, ROS levels were detected using the DCFH‐DA apoptosis detection kit according to the manufacturer’s instructions. The experimental groups included: H_2_O_2_‐treated group, blank control group, and GP‐treated groups (0.02%, 0.04%, 0.08%, and 0.16%). Cells from each well were collected into flow cytometry tubes, the medium was discarded, and cells were resuspended in PBS. Finally, the fluorescence intensity of each group was analyzed using flow cytometry.

### 2.6. Quantitative Real‐Time Polymerase Chain Reaction (qRT‐PCR) Experiments

RNA from B16 cells was extracted using Monzol reagent (Monad) and subsequently reverse transcribed into complementary DNA using a Reverse Transcription kit (Beyotime, China) following the manufacturer’s instructions. The qRT‐PCR was conducted on a real‐time fluorescence quantitative PCR instrument (Bio‐Rad CFX96^TM^, USA) with the BeyoFast SYBR qPCR kit (Beyotime Co., Ltd.). qRT‐PCR experiments were carried out to verify and compare the mRNA expression levels of melanin‐related genes microphthalmia‐associated transcription factor (MITF), tyrosinase (TYR), tyrosinase‐related protein 1 (TYRP1), and *β-*catenin in B16 cells under different treatments.

### 2.7. In Vivo Experiments

The pigmentation effects of GP under H_2_O_2_‐induced stress were investigated using mice as an in vivo model. All animal experiments were conducted in accordance with the guidelines approved by the Academic Ethics Committee of Anhui Normal University, and the ethics approval/permit number was AHNU‐ET 2022064. Thirty healthy male C57BL/6 mice, aged four weeks and weighing approximately 20 g, were kept in a temperature‐controlled environment with a 12‐h light/dark cycle and provided with unlimited access to food and water.

Prior to treatment, the dorsal skin of all mice was shaved to induce the transition of hair follicles from the telogen to the anagen stage. The 30 mice were then randomly allocated into six groups, each consisting of five mice. The first group received topical application of 200 μL of water at 9 a.m., serving as the CK group. The remaining five groups were treated with 200 μL of 3% H_2_O_2_ at 9 a.m. topically. Additionally, at 4 p.m. daily, the six groups were administered topically with 200 μL of water, 0.5% GP, 1% GP, 2% GP, and 1% 8‐methoxypsoralen (8‐MOP) (serving as the positive control), respectively. This treatment regimen was repeated once daily for a period of 30 days. Subsequently, the hair pigmentation and growth of the mice were assessed.

### 2.8. Statistical Analysis

Data were expressed as mean ± standard deviation (SD). Analysis of variance (ANOVA) was performed and visualized using GraphPad Prism software, followed by Dunnett’s test for pairwise comparisons. Statistical significance was defined as ^∗^ *p* < 0.05 and ^∗∗^ *p* < 0.01 compared to the control group.

## 3. Results

### 3.1. GP Protects B16 Cells Against H_2_O_2_‐Induced Cytotoxicity and Apoptosis

**FIGURE 1:**
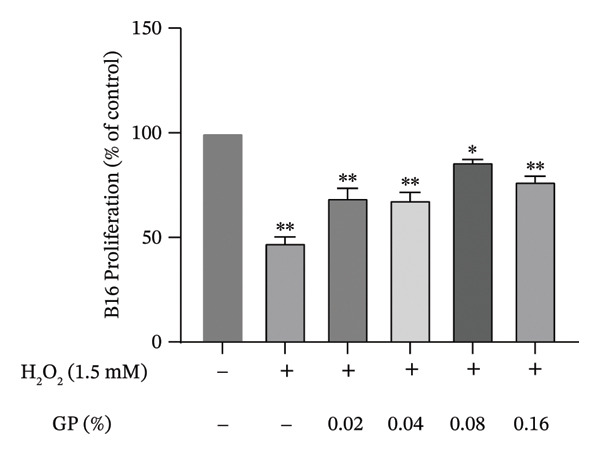
Effect of GP (0.02%–0.16%) on B16 cell viability after 1.5 mM H_2_O_2_ exposure for 30 min, assessed by CCK‐8. ^∗^ *p* < 0.05 and ^∗∗^ *p* < 0.01.

To investigate the protective effects of GP against H_2_O_2_‐induced oxidative stress, B16 cells were pretreated with varying concentrations of GP (0.02%–0.16%) for 24 h, followed by exposure to 1.5 mM H_2_O_2_ for 0.5 h. Cell viability and apoptosis were then assessed using the CCK‐8 assay. The specific concentrations and treatment durations for GP and H_2_O_2_ were selected based on our previous research (unpublished data). As shown in Figure 1, H_2_O_2_ exposure significantly increased the proportion of apoptotic cells in B16 cultures. However, the presence of GP substantially mitigated the cytotoxic effects induced by H_2_O_2_. Importantly, GP treatment alone at concentrations between 0.02% and 0.16% did not affect cell viability. These results highlight the protective potential of GP against H_2_O_2_‐induced oxidative stress in B16 cells.

### 3.2. Effects of GP on Melanin Content in B16 Cells Under H_2_O_2_‐Induced Oxidative Stress

**FIGURE 2:**
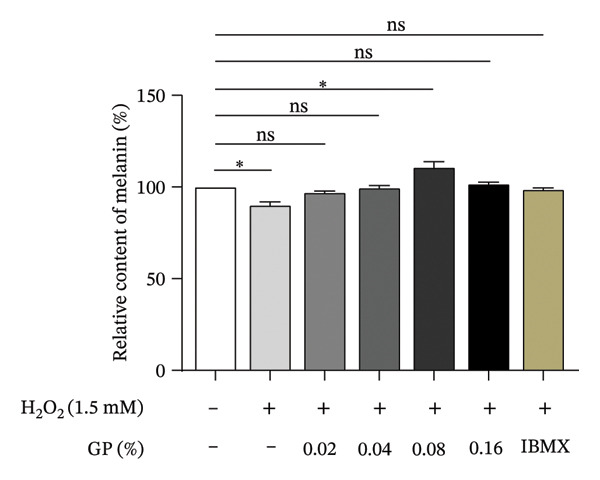
Melanin content quantified by the NaOH method and expressed as percentage relative to control (=100%). B16 cells were pretreated with 1.5 mM H_2_O_2_ for 30 min, followed by GP treatment (0.02%–0.16%) for 48 h. IBMX (35 μM) served as positive control. ^∗^ *p* < 0.05.

Before treatment with GP, B16 melanoma cells were treated with 1.5‐mM H_2_O_2_ for 30 min, and melanin content was measured. The level of melanin was expressed as a percentage relative to the control. We found that treatment with GP significantly increased melanin levels in a dose‐dependent manner, as shown in Figure 2. After treatment with H_2_O_2_, 0.02%, 0.04%, 0.08%, and 0.16% GP for 48 h, the melanin content compared with the control group was 90.32% (^∗^), 96.96% (no significant difference, NSD), 99.48% (NSD), 110.62% (^∗^), and 101.54% (NSD), respectively. Additionally, treatment with 3‐isobutyl‐1‐methylxanthine (IBMX) for 48 h resulted in melanin content of 98.60% (NSD). IBMX (35 μM) served as a positive pigment standard to promote melanin synthesis, while H_2_O_2_ (1.5 mM) served as a bleaching standard. As shown in the results, GP concentrations above 0.02% could counteract the effects of H_2_O_2_ on B16 melanoma cells, and higher concentrations (0.04%, 0.08%, and 0.16%) exhibited even stronger stimulatory effects on melanin formation compared with IBMX.

### 3.3. Effect of GP on Intracellular Tyrosinase and SOD Activity in B16 Cells Under H_2_O_2_‐Induced Oxidative Stress

**FIGURE 3:**
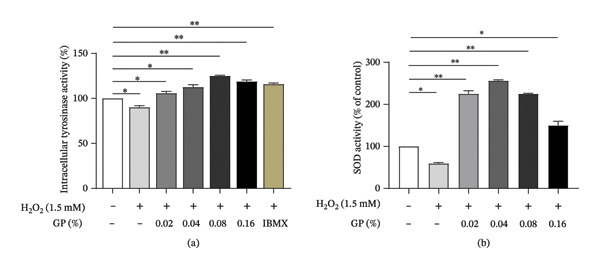
Effect of GP (0.02%–0.16%) on tyrosinase (a) and SOD (b) activity in B16 cells exposed to 1.5‐mM H_2_O_2_ for 30 min, followed by 48‐h GP treatment. Tyrosinase activity was determined using L‐tyrosine assay, and SOD activity expressed as percentage relative to control (=100%). ^∗^ *p* < 0.05 and ^∗∗^ *p* < 0.01.

Under hydrogen peroxide stress, treatment with GP significantly increased tyrosinase activity in B16 melanoma cells, as depicted in the graph. After 48 h of treatment with 0.02%, 0.04%, 0.08%, and 0.16% GP, compared with the control group (CK), the intracellular tyrosinase activity was 105.83% (^∗^), 112.66% (^∗^), 125.07% (^∗∗^), and 118.88% (^∗∗^), respectively. Additionally, cells treated with IBMX (35 μM) exhibited intracellular tyrosinase activity equivalent to 115.86% of the CK (^∗∗^). Hydrogen peroxide exerted an inhibitory effect on tyrosinase activity, with residual enzyme activity at 90.06% of the CK (^∗^). IBMX (35 μM) served as a positive pigment standard, while 1.5‐mM hydrogen peroxide served as a bleaching standard. The results demonstrate that concentrations of GP exceeding 0.02% can counteract the effects of hydrogen peroxide on B16 melanoma cells, with higher concentrations (0.08% and 0.16%) exhibiting even greater stimulatory effects compared to IBMX, as illustrated in Figure 3a.

SOD catalyzes the conversion of superoxide anions into hydrogen peroxide and oxygen, functioning as a vital antioxidant enzyme in biological systems. Reduced SOD activity indicates elevated H_2_O_2_‐induced oxidative stress levels within an organism. As illustrated in Figure 3b, the SOD levels in the hydrogen peroxide group were 59.4% compared with the control group (CK). In contrast, the GP‐treated groups showed SOD levels of 225.0%, 256.3%, 225.0%, and 150.0%, respectively. These results indicate that hydrogen peroxide treatment increases intracellular H_2_O_2_‐induced oxidative stress levels compared with the control, while GP concentrations above 0.02% can mitigate oxidative stress in B16 cells (Figure 3b).

### 3.4. Detection of the ROS Levels in B16 Cells by Flow Cytometry

**FIGURE 4:**
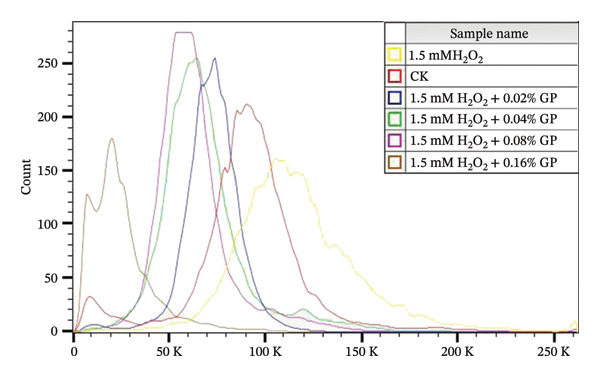
Detection of the ROS levels in B16 cells measured by DCFH‐DA flow cytometry. Cells were exposed to 1.5‐mM H_2_O_2_ for 30 min and treated with GP (0.02%–0.16%) for 48 h. Fluorescence intensity represents intracellular ROS levels.

The changes in intracellular ROS levels in B16 cells under H_2_O_2_‐induced oxidative stress with different concentrations of GP were detected using flow cytometry. B16 cells were first exposed to 1.5‐mM H_2_O_2_ for 30 min, followed by incubation in culture medium containing various concentrations of GP for 48 h. Figure 4 shows that ROS levels in cells increased significantly after H_2_O_2_ treatment compared with the control (CK) group. However, subsequent treatment with GP reduced intracellular ROS accumulation, as evidenced by a decrease in fluorescence intensity.

### 3.5. GP Modulates the Expression of Genes Related to Induced Melanin Synthesis

Studies have demonstrated the involvement of various signaling pathways in the regulation of melanocyte function and hair pigmentation. The Wnt/β‐catenin signaling pathway, for example, has been shown to play a critical role in melanocyte proliferation and differentiation. Dysregulation of this pathway can lead to abnormal hair pigmentation and premature graying. MITF is a master regulator of melanocyte development and function, controlling the expression of key melanogenic enzymes such as tyrosinase. Dysregulation of MITF expression or activity can lead to aberrant melanocyte function and contribute to hair graying [8, 19].

**FIGURE 5:**
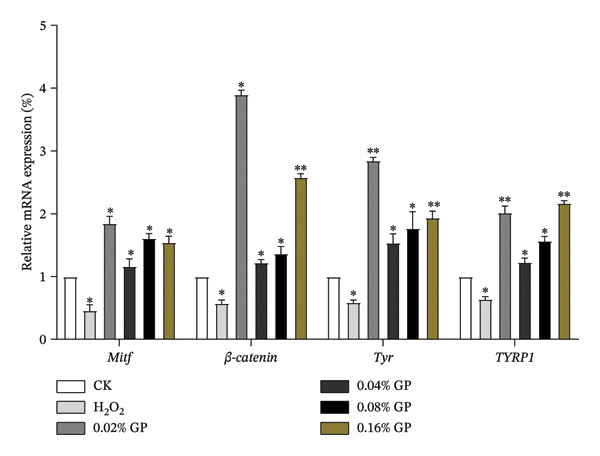
Effect of GP on the expression of melanogenic genes assessed by qRT‐PCR and normalized to control, ^∗^ *p* < 0.05 and ^∗∗^ *p* < 0.01.

The expression of genes involved in melanin synthesis and regulation was examined using qRT‐PCR. As shown in Figure 5, after treatment with hydrogen peroxide for 30 min, the expression of synthesis‐related genes MITF, TYR, TYRP1, and *β-*catenin in B16 cells showed a noticeable decrease. However, compared with the control group, GP treatment significantly increased the expression of these genes. These findings indicate that GP‐induced pigmentation occurs through the upregulation of MITF and TYR family genes and suggest that GP may modulate melanin synthesis through the Wnt/β‐catenin signaling pathway.

### 3.6. Pigmentation of Mice Hair

**FIGURE 6:**
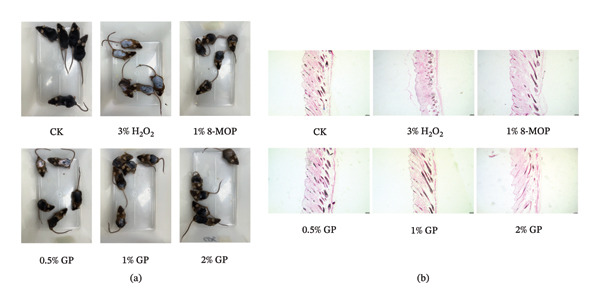
Effects of GP on hair pigmentation and follicle morphology in vivo. Mice were treated with 3% H_2_O_2_ topically at 9 a.m. daily, followed by topical GP (0.5%, 1%, and 2%) or 1% 8‐MOP at 4 p.m. for 30 days. (a) Representative images of hair pigmentation. (b) HE staining showing hair follicle length.

Photographic techniques were employed to assess and compare hair growth and pigmentation in mice treated with GP. In the hydrogen peroxide group, newly grown hair appeared yellow, indicating depigmentation of hair follicles under hydrogen peroxide stress. In the GP/H_2_O_2_ group or the positive control group treated with 8‐MOP, black hair growth was observed, suggesting that GP can counteract hydrogen peroxide‐induced depigmentation. Moreover, mice in the GP treatment group not only exhibited hair darkening but also showed faster growth (Figure 6a).

To further investigate the effect of GP on hair growth, Hematoxylin–Eosin (HE) staining was performed to observe the length of hair follicles. On the 30th day after depilation, the hair follicle length of mice in the blank group, MOP/H_2_O_2_ positive control group, and GP/H_2_O_2_ group were all significantly higher than that of the hydrogen peroxide group mice (Figure 6b). Overall, GP demonstrated the ability to resist the inhibitory effect of H_2_O_2_ on hair growth.

## 4. Discussion

Our study provides compelling evidence that GP extract effectively mitigates H_2_O_2_‐induced oxidative stress in melanocytes and prevents hair graying both in vitro and in vivo. The findings support our initial hypothesis that GP, renowned for its potent antioxidant properties, could protect melanocytes from oxidative damage—a key driver of age‐related hair graying [10, 11]. The protective effects of GP observed in B16 cells, including enhanced viability, reduced apoptosis, and decreased ROS levels, are consistent with its well‐documented antioxidant capacity [13, 17]. Notably, GP not only neutralized oxidative stress but also actively promoted the melanogenic functions of surviving melanocytes. The upregulation of melanin synthesis, tyrosinase activity, and key genes like MITF, TYR, and TYRP1 suggests that GP acts beyond mere cytoprotection, directly stimulating the pigmentation machinery.

Among its diverse constituents, GP is particularly rich in gypenosides (triterpenoid saponins), which have been extensively reported to possess strong antioxidant, anti‐inflammatory, and neuroprotective activities [13, 17]. We postulate that it is these gypenosides that are primarily responsible for the observed effects. Their antioxidant nature directly correlates with the reduction in ROS and increase in SOD activity. Furthermore, some saponins have been shown to modulate various signaling pathways, including MAPK and AKT [17], which could crosstalk with or influence the Wnt/β‐catenin pathway observed here.

Beyond its direct antioxidant function, GP may exert multitargeted protective actions relevant to hair pigmentation. Previous studies have shown that gypenosides display anti‐inflammatory effects by suppressing NF‐κB potential influence, antiapoptotic activity by modulating Bcl‐2 family proteins, and mitochondrial protective properties [17]. These pleiotropic mechanisms correspond well with our findings that GP not only lowered ROS levels but also enhanced melanocyte survival, increased SOD activity, and may potentially influence the Wnt/β‐catenin pathway. Thus, GP may represent a holistic approach to counteracting hair graying by simultaneously mitigating oxidative stress, preserving cellular integrity, and supporting melanogenic activity.

Interestingly, the bioactivity of gypenosides shows notable similarities to other natural compounds studied for hair pigmentation, such as ginsenosides from *Panax ginseng* and polyphenols from tea (*Camellia sinensis*). Both ginsenosides (e.g., as demonstrated in a study on red ginseng extract for hair growth [20] and tea polyphenols [21, 22]) have been reported to protect melanocytes against oxidative damage and regulate melanogenic pathways. However, gypenosides are more abundant and structurally diverse in GP, which may confer broader biological activity. While tea polyphenols primarily act through direct antioxidant capacity and Nrf2 pathway modulation, gypenosides additionally modulate Wnt/β‐catenin and MAPK signaling [17]. This comparison situates our findings within a broader context and highlights the potential comparative advantages of GP extract.

Future studies focusing on the isolation of specific gypenosides from our extract will be essential to pinpoint the exact molecule(s) driving the antigraying benefits.

## 5. Limitations and Future Perspectives

While our study provides promising evidence for the antigraying potential of GP extract, several limitations should be acknowledged. First, the evidence for the modulation of the Wnt/β‐catenin signaling pathway, while suggestive, remains indirect, as it is based solely on gene expression changes (β‐catenin and MITF). Definitive confirmation through pathway inhibition assays, reporter gene analysis, or Western blotting for *β*‐catenin protein nuclear translocation is required in future work. Second, our in vivo experiments were conducted with a relatively small sample size (*n* = 5 per group) and utilized only male C57BL/6 mice. This limits the generalizability of our findings and does not account for potential sex‐based differences in the response to oxidative stress or GP treatment. Future studies should employ larger, mixed‐sex cohorts to strengthen the statistical power and broaden the applicability of the results.

Looking forward, several key directions emerge from this work. The primary task is the isolation and identification of the specific bioactive gypenoside(s) within the GP extract responsible for the observed antioxidant and melanogenic effects. Elucidating their precise molecular targets and downstream signaling cascades—beyond the preliminary link to Wnt/β‐catenin—will be crucial. Furthermore, translating these promising preclinical results into clinical efficacy and safety is essential. Well‐designed randomized controlled trials are needed to evaluate the topical or oral application of GP or its active constituents for preventing or treating hair graying in humans. Finally, exploring potential synergistic effects with other known antioxidants or hair‐growth promoters could lead to the development of more potent multitarget formulations.

## 6. Conclusion

In conclusion, while acknowledging the preliminary nature of some mechanistic evidence and the scale of the in vivo study, our study provides compelling evidence that GP extract is a potent natural candidate against oxidative stress–induced hair graying. We demonstrated that GP robustly protects melanocytes from H_2_O_2_‐induced damage by (1) enhancing cellular antioxidant defense (e.g., increasing SOD activity), (2) significantly reducing ROS accumulation, (3) upregulating key melanogenic genes (MITF, TYR, and TYRP1) and tyrosinase activity, and 4) effectively preserving hair follicle pigmentation and promoting hair growth in a mouse model. These multi‐faceted actions, particularly the potential involvement of the Wnt/β‐catenin pathway, underscore GP’s capacity to not only neutralize oxidative damage but also actively stimulate the pigmentation machinery.

These findings firmly establish GP’s therapeutic potential for preventing and potentially treating oxidative stress–related hair graying. Given its longstanding use as a safe traditional herbal medicine, GP holds strong promise for development into a novel, evidence‐based natural therapy for hair graying and related dermatological conditions.

## Funding

This study was funded by Wuhu Sci‐Tech Program Project, 2024KJ002.

## Conflicts of Interest

The authors declare no conflicts of interest.

## Data Availability

The data that support the findings of this study are available from the corresponding author upon reasonable request.
